# Caffeine Promotes Global Spatial Processing in Habitual and Non-Habitual Caffeine Consumers

**DOI:** 10.3389/fnhum.2013.00694

**Published:** 2013-10-17

**Authors:** Grace E. Giles, Caroline R. Mahoney, Tad T. Brunyé, Holly A. Taylor, Robin B. Kanarek

**Affiliations:** ^1^Department of Psychology, Tufts University, Medford, MA, USA

**Keywords:** caffeine, arousal, spatial memory, global, local

## Abstract

Information processing is generally biased toward global cues, often at the expense of local information. Equivocal extant data suggests that arousal states may accentuate either a local or global processing bias, at least partially dependent on the nature of the manipulation, task, and stimuli. To further differentiate the conditions responsible for such equivocal results we varied caffeine doses to alter physiological arousal states and measured their effect on tasks requiring the retrieval of local versus global spatial knowledge. In a double-blind, repeated-measures design, non-habitual (Experiment 1; *N* = 36, *M* = 42.5 ± 28.7 mg/day caffeine) and habitual (Experiment 2; *N* = 34, *M* = 579.5 ± 311.5 mg/day caffeine) caffeine consumers completed four test sessions corresponding to each of four caffeine doses (0, 100, 200, 400 mg). During each test session, participants consumed a capsule containing one of the three doses of caffeine or placebo, waited 60 min, and then completed two spatial tasks, one involving memorizing maps and one spatial descriptions. A spatial statement verification task tested local versus global spatial knowledge by differentially probing memory for proximal versus distal landmark relationships. On the map learning task, results indicated that caffeine enhanced memory for distal (i.e., global) compared to proximal (i.e., local) comparisons at 100 (marginal), 200, and 400 mg caffeine in non-habitual consumers, and marginally beginning at 200 mg caffeine in habitual consumers. On the spatial descriptions task, caffeine enhanced memory for distal compared to proximal comparisons beginning at 100 mg in non-habitual but not habitual consumers. We thus provide evidence that caffeine-induced physiological arousal amplifies global spatial processing biases, and these effects are at least partially driven by habitual caffeine consumption.

## Introduction

The way we perceive our environment has implications for our ability to attend to environmental cues and successfully navigate from one point to another. Visual perception research suggests that information processing shows a global precedence, with processing beginning at global levels and then progressing to relatively local levels (Navon, 1977, 2003; Kimchi, 1992), even as early as infancy (Cassia et al., 2002). Some recent findings suggest that this global precedence is not a stable trait but rather subject to change under conditions of emotional and physiological arousal. The directionality of these changes, however, remains under debate; indeed some studies find evidence of arousal-induced global processing advantages, and yet others find the opposite. Further, the generalizability of these effects across information types has not been fully explored. Several studies have examined these issues with visual perception and verbal memory, but would they also carry over to the processing and mental representation of relatively real-world spatial information? Toward further elucidating the directionality and breadth of arousal effects on local versus global processing, the present research examines whether physiological arousal states modulate global precedence on tasks involving the processing, representation, and retrieval of spatial information. Below we review extant literature related to arousal influences on perception and memory, and then briefly review emerging evidence related to caffeine’s influence on these processes as a basis for motivating our manipulation and hypotheses.

### Arousal and memory

Equivocal extant data suggests that arousal states may accentuate either a local or global processing bias, at least partially dependent on the nature of the manipulation, task, and stimuli. Emotional information may be conceptualized in two orthogonal dimensions: valence (positive or negative) and arousal (high or low), which utilize somewhat distinct neural processes (Kensinger and Corkin, 2004).

Two different research methods have been used to determine the relationship between emotional state and global/local processing biases, the first of which investigates memory and attention to emotional stimuli. Exposure to emotional stimuli generally narrows attention and impairs memory for details peripheral to the salient image features (Loftus, 1979; Loftus and Burns, 1982). At the same time, memory for certain details, mainly the central details (e.g., a weapon), are enhanced in arousing relative to neutral scenes (Kensinger and Schacter, 2006, 2007). Enhanced memory for central, emotionally arousing elements of a scene is not necessarily due to increased attention for those elements; this enhancement is present after as little as one eye fixation (Christianson et al., 1991). These findings are generally aligned with Easterbrook’s (1959) seminal arousal hypothesis, which proposed that heightened emotional arousal reduces the range of cues an individual uses to gather information from ongoing events (Easterbrook, 1959).

The second method entails experimentally inducing emotional states, and assessing the influence on memory and attention to neutral stimuli. Emotional states may act as information cues, i.e., *affect as information* approach (Schwarz and Clore, 1983; Clore and Palmer, 2009). Gasper and Clore (2002) proposed the *levels of focus* approach, in which mood states differentially guide attention to global versus local cues. Individuals in sad emotional states are less likely to attend to global perceptual cues than individuals in happy emotional states (Gasper and Clore, 2002); specifically, when asked to indicate whether a target figure is most similar to a figure that matches either the local or global features of the target figure, participants showed a marked tendency to match on global features when in a positive versus negative or neutral mood. Similar results were found with verbal stimuli: when participants were placed in a positive versus negative mood they tended to falsely recall a higher proportion of highly associated but never presented words, i.e., critical lures, suggesting increased “gist” or global verbal associative processing (Storbeck and Clore, 2005).

Other studies suggest that arousal, rather than valence, may be responsible for at least some of the above findings. For instance, Corson and Verrier (2007) argue that earlier verbal memory results can be attributed to arousal alone rather than accompanying valence (Corson and Verrier, 2007); specifically, when valence was held constant, high arousal states showed the global verbal associative processing found by Storbeck and Clore (2005). Arousal also influences the ability to switch between local and global attentional focuses, as trained soccer players have impaired local attention relative to non-athletes, but are better able to switch from local to global perspectives during periods of high physiological arousal (Pesce et al., 2007). More chronic arousal, such as that experienced by individuals with post-traumatic stress disorder (PTSD) with heightened basal arousal levels, also leads to global processing biases (Vasterling et al., 2004).

Whether emotional states or cues contribute to a global or local focus may be driven, in part, by their motivational intensity. Motivational intensity as defined by Gable and Harmon-Jones (2010) is the “impetus to act.” They propose that within positive affective states, low approach motivation (e.g., content) broadens attentional scope whereas high approach motivation (e.g., enthusiastic) narrows attentional scope (Harmon-Jones and Gable, 2008). The same has been found within negative affective states, as viewing a series of images low in approach intensity (i.e., sad) accentuated global attention whereas viewing a series of images high in approach intensity (i.e., disgusting) reduced global attention relative to neutral images (Gable and Harmon-Jones, 2010). Such results are likely not attributable to differences in arousal, as viewing images characterized as negative in affect and high in arousal and approach motivation (i.e., appetitive desserts) narrowed attentional scope, whereas pedaling on a bicycle, which increases cardiovascular arousal but does not affect or approach motivation, had no influence on attentional scope (Gable and Harmon-Jones, 2013).

Thus, there is converging evidence that arousal states (with or without specific valence or motivational attributes) may influence both the processing and representation of abstract shapes (e.g., Gasper and Clore, 2002) and word lists (e.g., Storbeck and Clore, 2005; Corson and Verrier, 2007). Further, when individuals attend to affectively salient images there appears to be an arousal-related increase in local processing (Loftus, 1979; Kensinger and Schacter, 2006); in contrast, when individuals attend to affectively neutral information while in a heightened arousal state there appears to be an increase in global processing.

Some recent research specifically asked whether the valence or arousal accompanying affective states modulated local versus global processing of spatial information. Brunyé et al. (2009) manipulated subjects’ emotional states by crossing arousal (high versus low) with valence (happy versus sad) and assessed memory for landmark relationships. They found that high arousal augmented global spatial processing, such that accuracy and response time for distal relative to proximal landmark judgments were higher and faster (respectively) for individuals in high relative to low arousal states, regardless of positive or negative valence (Brunyé et al., 2009). This adds converging evidence to suggest that heightened arousal, regardless of valence, promotes global processing advantages with map-based spatial information.

### Caffeine, arousal, and global processing

The research reviewed above highlights the importance of selectively targeting arousal mechanisms without altering the valence or motivational intensity of subjective mood states; in the behavioral paradigms frequently used in laboratory settings, this can be difficult. Indeed the pictures, music, and/or autobiographical recall instructions typically selected for mood induction tend to be selected to specifically induce single affective states such as happiness, sadness, anger, or fear (Lang et al., 1993, 1998; Husain et al., 2002; Jallais and Gilet, 2010). Indeed it is difficult to imagine images or music that can induce a heightened arousal state without also being associated with anger, anxiety, fear, or excitement; similarly, it is difficult to induce a suppressed arousal state without it being associated with sadness or a positive state of relaxation/contentment.

A relatively selective approach to influencing arousal states is to manipulate subjective and physiological arousal by administering a psychostimulant such as caffeine. Caffeine is the most common behaviorally active substance in the world. Almost 90% of the individuals in the United States consume caffeine, and daily caffeine intake averages approximately 200 mg/day (Frary et al., 2005; Smith, 2011). Habitual caffeine consumption may stem, in part, from its perceived beneficial effects on arousal and vigilance (Nehlig et al., 1992b; Lieberman, 2001). Biochemically, caffeine consumption results in increased dopamine and serotonin, which have been linked to the enhancement of processes that require executive control (Ferre et al., 1997; Abrams et al., 2005; Brunyé et al., 2010b; Mahoney et al., 2011). In addition, caffeine consumption has been shown to increase cortisol, an index of physiological arousal (Lovallo et al., 2005), and enhance alertness, vigilance, and psychomotor performance (Lieberman, 2003). Other cognitive influences of caffeine, including attention, depend more on environmental factors such as sleep deprivation as well as individuals’ habitual caffeine intake (Rogers et al., 2005; Brunyé et al., 2010a,b).

Caffeine reliably increases arousal in habitual and non-habitual caffeine consumers (Childs and de Wit, 2006). Similarly, caffeine withdrawal reduces arousal (i.e., lower rated vigor and higher rated fatigue) in habitual consumers (Lane, 1997; Lane and Phillips-Brute, 1998; Haskell et al., 2005). However, evidence is mixed as to whether caffeine influences affective valence. Caffeine increased anxiety and tension, but only at a high dose (Childs and de Wit, 2006; Mahoney et al., 2011), but did not influence anxiety or hedonic tone in habitual and non-habitual caffeine consumers (Smith et al., 2006a). Caffeine withdrawal may also influence affective valence, as high habitual caffeine consumers reported feeling less vigorous and more angry, confused, depressed, and fatigued after abstaining from their normal caffeine intake than after consuming caffeine *ad libitum* (Lane, 1997; Lane and Phillips-Brute, 1998). Thus caffeine reliably increases arousal but not affective valence, but the caffeine withdrawal from overnight abstinence could partially account for mixed findings in high habitual caffeine consumers.

Recent research suggests that caffeine accentuates global processing biases in both visual perception (Mahoney et al., 2011) and language-based tasks (Brunyé et al., 2012). Mahoney et al. (2011) administered a range of caffeine doses (0–400 mg) and asked participants to complete two visual attention tasks, i.e., the Hierarchical Shape Task (Kimchi and Palmer, 1982) and Hierarchical Letter Task (Navon, 1977). Individuals responded faster to global relative to local comparisons, and this effect became pronounced with caffeine administration. Brunyé et al. (2012) used the same dose-ranging design, and used a language tasks that required subjects to identify and correct errors in an extended text. Caffeine enhanced error detection rates for global (e.g., subject-verb agreement errors), but not local (e.g., spelling errors) elements of the text beginning at 200 mg in non-habitual caffeine consumers and at 400 mg in habitual caffeine consumers.

### Present study

The primary aim of the present study is to assess the influence of arousal on global versus local spatial memory, in order to better understand the relationship between arousal and memory for “gist” and detail information without the potential confounding influence of emotional valence. The extant literature provides a strong basis for generating hypotheses regarding caffeine’s influence on the processing and representation of local versus global spatial information. A number of studies have suggested that encoding of spatial information is an automatic rather than effortful process (Hasher and Zacks, 1979; Ellis, 1990; Andrade and Meudell, 1993) and thus unlikely to be influenced by arousal states; other studies, however suggest the opposite (Light and Zelinski, 1983; Arbuckle et al., 1994; Kessels et al., 2005). Indeed a growing body of evidence suggests that the ability to accurately process and mentally represent spatial information is contingent upon several factors such as goals, affective states, working memory load, and strategies (McNamara et al., 1992; Taylor et al., 1999; Waller, 2000; Hegarty et al., 2006; Brunye and Taylor, 2008; Maddox et al., 2008; Brunyé et al., 2009; Gyselinck et al., 2009; Meneghetti et al., 2009; Gardony et al., 2011). Thus, a number of studies suggest that there is limited automaticity to the encoding of spatial location information, although it may be processed less effortfully than some other types of information (Thomas et al., 2012). Our first hypothesis, therefore, is that the arousal states produced via caffeine administration will influence participants’ ability to accurately memorize spatial information.

The effect of caffeine on spatial memory is expected to manifest specifically when assessing memory for local versus global details of a spatial scene. Previous work suggests that arousal but not valence enhances memory for global spatial relationships (Brunyé et al., 2009) and that caffeine amplifies global processing biases (Mahoney et al., 2011). To examine whether these results hold for caffeine and spatial stimuli, we assess participants’ ability to make inferences about proximal (two landmarks close to one another) versus distal (two landmarks far from one another) spatial relationships after consuming one of four caffeine doses. Given earlier findings, our second hypothesis states that increasing doses of caffeine will induce an increasingly global focus in spatial memory.

Finally, because chronic caffeine consumption can increase adenosine receptor density in the brain (Daval et al., 1989; Rudolphi et al., 1989; Varani et al., 1999) and influence necessary doses required to achieve cognitive effects (Evans and Griffiths, 1992; Jacobson and Thurman-Lacey, 1992; Lyvers et al., 2004; Attwood et al., 2007; Brunyé et al., 2010a, 2012), consumption patterns may modulate our hypothesized effects. To address this issue, we separately recruited participants who rarely (Experiment 1) or regularly (Experiment 2) consume caffeine. Our final hypothesis states that the influence of caffeine on spatial memory will be evident at lower doses in low habitual caffeine consumers than in high habitual consumers.

## Materials and Methods

### Participants

Thirty six undergraduate students who were low habitual caffeine consumers (less than 100 mg/day, *M* = 42.45 ± 28.68 mg/day) participated in Experiment 1 and 34 students who were high habitual caffeine consumers (at least 300 mg/day, *M* = 579.51 ± 311.48 mg/day) participated in Experiment 2 (see Table 1). Students participated for monetary compensation ($10 USD/h). All students were non-nicotine users, in good health, and did not use prescription medication other than oral contraceptives. Written informed consent was obtained, and all procedures were jointly approved by the Tufts University Institutional Review Board and the Human Use Review Committee of the U.S. Army Research Institute for Environmental Medicine.

**Table 1:** **Age, gender, BMI, and caffeine intake distribution for study subjects**.

Habitual caffeine intake	n (female)	Age	BMI	Caffeine intake
	
		M ± SD	M ± SD	M ± SD
Low (<100 mg/day)	36 (20)	19.08 ± 1.32	23.15 ± 3.01	42.45 ± 28.68
High (>300 mg caffeine/day)	34 (26)	20.00 ± 1.46	22.65 ± 4.64	579.51 ± 311.48

### Design

Both Experiment 1 and Experiment 2 used a double-blind, repeated-measures design with four levels of caffeine (0, 100, 200, 400 mg caffeine). The highest dose of caffeine approximates that found in a 20 oz coffee portion served at a major franchise coffee house (i.e., 415 mg; www.starbucks.com). Caffeine order was counterbalanced across participants. In order to control for taste, caffeine or placebo was administered in capsule form; capsules were identical in color, size, weight, and shape. The caffeine was 99.8% pure anhydrous USP-grade powder. Placebo capsules were filled with physiologically inert microcrystalline cellulose powder, which was also used as filler material in the two lower-dose caffeine capsules.

### Questionnaires and cognitive tasks

#### Brief mood introspection scale

The Brief Mood Introspection Scale (BMIS) involves rating current mood state in accordance with 16 adjectives (8 positive and 8 negative) on a series of 4 point Likert scales anchored at 1 (definitely do not feel) and 4 (definitely feel) (Mayer and Gasche, 1988). The BMIS was factored into four subscales: pleasant, unpleasant, arousal and calm, and served as a manipulation check to ensure that caffeine increased feelings of arousal but did not reliably alter feelings of positive or negative affect.

#### Map learning task

Four maps were adapted from Grinnell, St. Olaf’s, and Occidental campus maps (i.e., Brunye et al., 2007). Each map was standardized to include 14 labeled buildings, 6 labeled roads, and a compass rose. Participants had 5 min to study a map, which was followed by a brief distraction task (i.e., simple arithmetic calculations) and then a spatial statement verification task. The statement verification task involved 56 sentences describing the relative spatial location between map locations (e.g., The Psychology Building is west of Anderson Hall) across two comparison distances (28 proximal, 28 distal). Participants responded “true” or “false” and dependent measures include accuracy and response time.

#### Spatial description task

This task followed the same procedure as the map learning task with the exception that participants read a description of an environment, instead of studying a map. Four sets of text were adapted from (Taylor and Tversky, 1992, see also Brunye and Taylor, 2008; Brunye et al., 2008). Each set of texts described an environment that included 7–10 landmarks. Participants had approximately 5 min to study the description, followed by the brief arithmetic distracter task and then the statement verification task. Dependent measures include accuracy and response time.

### Procedure

Participants completed one practice session and all four caffeine conditions on separate days, resulting in five test sessions. There was a minimum three day wash-out period between test sessions. Participants were instructed not to eat or drink anything (with the exception of water) after 9:00 p.m. the night before a test session and not to use any over-the-counter medications or herbal supplements 24 h prior to testing. A 12-h abstinence period is thought to be a sufficient wash-out period to attenuate the effects of earlier caffeine consumption, given that the mean plasma and elimination half-life of caffeine ranges from 3 to 10 h (Blanchard and Sawers, 1983; Scott et al., 1989; Nehlig et al., 1992a). Test sessions began between 7:00 and 9:30 a.m.

When participants arrived in the morning, they consumed a capsule containing one of the three doses of caffeine or placebo along with a cup of water. Sixty minutes after consuming the capsule, participants completed the BMIS, map task, and spatial description task, in the same order within-participants and counterbalanced order across participants. Timing of testing was based on previous research showing that caffeine peak plasma concentrations vary between individuals and occur between 30 and 120 min after consumption (Blanchard and Sawers, 1983; Arnaud, 1987; Smith, 2002).

### Statistics

The BMIS was analyzed using an Analyses of Variance (ANOVA) with Caffeine condition (0, 100, 200, 400 mg) and Subscale (Positive, Negative, Arousal, Calm) as within-participants factors. The map task and spatial descriptions tasks were analyzed using an ANOVA with Caffeine condition (0, 100, 200, 400) and test Distance (proximal, distant) as the within-participants factors. Dependent measures include response time and accuracy. An effect was deemed statistically significant if the likelihood of its occurrence by chance was *p* < 0.05. When sphericity was violated, Greenhouse–Geisser corrected *p*-values were used. When an ANOVA yielded a significant main effect, *post hoc* tests using the Bonferroni correction were conducted. All statistical analyses were performed using SPSS 12.0.

## Results

### Manipulation check

In low habitual caffeine consumers, analysis of BMIS data indicated main effects of Subscale *F*(3, 105) = 155.094, *p* < 0.001 (η^2^ = 0.714), and marginal effects of Caffeine, *F*(3, 105) = 2.251, *p* < 0.09 (η^2^ = 0.003); these effects were qualified by an interaction between Subscale and Caffeine, *F*(9, 315) = 2.262, *p* < 0.05 (η^2^ = 0.004). Follow-up analyses demonstrated that caffeine did not influence rated positive (*p* > 0.33), negative (*p* > 0.17), or calm mood (*p* > 0.26) but increased rated arousal *F*(3, 105) = 4.882, *p* < 0.01 (η^2^ = 0.310). Paired *t*-tests showed that rated arousal was marginally higher after 200 mg *t*(35) = 1.858, *p* < 0.08 (*d* = 0.310) and significantly higher after 400 mg caffeine *t*(35) = 3.388, *p* < 0.01 (*d* = 0.565) than placebo (Table 2).

**Table 2:** **Brief mood introspection scale (BMIS)**.

		Pleasant		Unpleasant		Arousal		Calm	
		M	SE	M	SE	M	SE	M	SE
Low habitual caffeine consumer	0 mg	20.06	0.82	23.58	0.69	12.31	0.36	13.11	0.27
	100 mg	21.06	0.85	24.61	0.73	12.78	0.44	13.11	0.35
	200 mg	21.22	0.78	24.36	0.69	13.28	0.50	13.19	0.29
	400 mg	20.64	0.85	23.78	0.67	14.28	0.52	13.64	0.26
High habitual caffeine consumer	0 mg	20.44	0.66	22.91	0.71	12.59	0.49	12.71	0.29
	100 mg	20.68	0.79	23.65	0.73	12.76	0.50	12.85	0.29
	200 mg	21.09	0.70	23.59	0.73	13.44	0.49	13.24	0.35
	400 mg	21.06	0.66	22.91	0.70	13.76	0.45	13.47	0.29

Paired *t*-tests demonstrated that 200 and 400 mg caffeine resulted in higher feelings of arousal (arousal-calm) than placebo (*p*’s < 0.01), but did not influence valence ratings (pleasant-unpleasant).

In high habitual caffeine consumers, main effects of Subscale *F*(3, 99) = 115.827, *p* < 0.001 (η^2^ = 0.707), and marginal effects of Caffeine, *F*(3, 99) = 2.267, *p* < 0.09 (η^2^ = 0.003) were not qualified by a Subscale by Caffeine interaction (*p* > 0.26). Thus 200 and 400 mg caffeine increased feelings of arousal, but not valence, in low habitual caffeine consumers, and such doses did not influence mood in high consumers.

### Caffeine order

Caffeine order was counterbalanced across participants to circumvent order effects. Nonetheless, all measures were subjected to analyses testing whether the first dose received, either low (i.e., 0 or 100 mg) or high (i.e., 200 or 400 mg) influenced results. In low habitual caffeine consumers, a low versus high first dose did not impact the BMIS (*p*’s > 0.13) or spatial descriptions task (*p*’s > 0.15). On the map learning task, no effects were found for reaction time (*p*’s > 0.16) but a marginal Caffeine by First Dose interaction on accuracy *F*(3, 102) = 2.262, *p* < 0.09 (η^2^ = 0.029) showed that accuracy was higher after 200 and 400 mg caffeine (*p*’s < 0.05) relative to placebo when the low dose was given first *F*(3, 18) = 2.859, *p* < 0.05 (η^2^ = 0.068) but no differences in accuracy when the high dose was given first (*p* > 0.59).

In high habitual caffeine consumers, a low versus high first dose did not impact the BMIS (*p*’s > 0.13). On the map learning task, no effects were found for accuracy (*p*’s > 0.43) but a marginal effect of First Dose on reaction time *F*(1, 30) = 3.017, *p* < 0.1(η^2^ = 0.189) in which reaction time was higher overall when subject were given the high relative to low dose first. On the spatial descriptions task, no effects were found for accuracy (*p*’s > 0.28). A Distance by First Dose interaction was found on reaction time *F*(1, 32) = 4.285, *p* < 0.05 (η^2^ = 0.028), but showed no differences between distal and proximal landmarks when either the low (*p* > 0.19) or high (*p* > 0.13) dose was given first. Thus caffeine order exerted only marginal effects on results.

### Experiment 1: Non-habitual caffeine consumers

#### Map learning task

Accuracy data replicated earlier results with a main effect of Distance *F*(1, 35) = 82.45, *p* < 0.001(η^2^ = 0.200), in which accuracy was higher for distant relative to proximal distances (Mean ± SEM Proximal = 0.77 ± 0.02; Distant = 0.89 ± 01). There was also a Distance × Caffeine interaction *F*(2.313, 80.962) = 4.695, *p* < 0.01 (η^2^ = 0.027). As shown in Table 3, verification accuracy rates for distant landmarks increased as a function of caffeine dose; specifically, for distant landmarks *F*(3, 105) = 7.072, *p* < 0.001 (η^2^ = 0.168) accuracy was marginally higher at 100 mg versus Placebo *t*(35) = 1.920, *p* < 0.07 (*d* = 0.32) and showed higher accuracy at 200 mg versus Placebo *t*(35) = 2.912, *p* < 0.01 (*d* = 0.485), and 400 mg versus Placebo *t*(35) = 4.333, *p* < 0.001 (*d* = 0.722). This same effect was not found when verifying landmarks close together (*p*’s > 0.65). These results replicate the symbolic distance effect, showing greater accuracy for landmarks that are farther apart than closer together, and show that caffeine amplifies the effect beginning at 100 mg caffeine intake.

**Table 3:** **Experiment 1 map learning task and spatial description task mean accuracy and response time in low habitual caffeine consumers (*n* = 36)**.

		Map learning task				Spatial descriptions task			
		Proximal		Distal		Proximal		Distal	
		M	SE	M	SE	M	SE	M	SE
Accuracy	0 mg	0.78	0.03	0.84	0.02	0.73	0.03	0.76	0.03
	100 mg	0.79	0.03	0.88	0.02	0.71	0.04	0.87	0.03
	200 mg	0.76	0.03	0.92	0.02	0.75	0.03	0.89	0.02
	400 mg	0.78	0.03	0.93	0.01	0.72	0.04	0.92	0.01
Response time	0 mg	3686.95	220.98	2938.86	168.88	2918.88	258.75	3100.91	294.12
	100 mg	3930.92	205.45	3205.35	161.19	3210.21	362.67	3233.22	276.08
	200 mg	3729.28	221.54	3089.34	180.49	2933.97	384.31	2948.43	344.09
	400 mg	3714.59	204.46	2999.88	157.26	2909.13	291.77	2830.50	270.01

The table represents proximal relative to distant distal comparisons for each of the four Caffeine doses (0, 100, 200, 400 mg). In the map learning task, within the distant condition accuracy was not higher at 100 mg versus Placebo, but showed higher accuracy at 200 mg versus Placebo (*p* < 0.01), and 400 mg versus Placebo (*p* < 0.001). This same effect was not found in the proximal condition. In the spatial description task, distal distance accuracy rates increased as a function of caffeine dose; specifically, within the distant condition accuracy was higher at 100 mg versus Placebo (*p* < 0.001), 200 mg versus Placebo (*p* < 0.01), and 400 mg versus Placebo (*p* < 0.001). This same effect was not found in the proximal condition.

Analysis of response time showed main effects for Distance *F*(1, 35) = 94.139, *p* < 0.001 (η^2^ = 0.241), in which response time was lower for distant relative to proximal landmarks, again replicating the symbolic distance effect (Proximal = 3765.43 ± 185.07 ms; Distant = 3058.36 ± 140.87 ms). No effects of Caffeine or interactions were found for response time.

#### Spatial description task

Accuracy data replicated earlier results with a main effect of Distance *F*(1, 34) = 85.421, *p* < 0.001 (η^2^ = 0.139), in which accuracy was higher for distant relative to proximal distances (Proximal = 0.73 ± 0.02; Distant = 0.86 ± 01). A marginal effect of Caffeine *F*(3, 102) = 2.227, *p* < 0.100 (η^2^ = 0.031) showed that accuracy did not differ from placebo after 100 mg caffeine, but was higher after 200 mg and marginally higher after 400 mg caffeine. As with the map task, there was also a Distance × Caffeine interaction *F*(2.382, 80.972) = 3.556, *p* < 0.05 (η^2^ = 0.029). As shown in Table 3, distant trial accuracy rates increased as a function of caffeine dose *F*(3, 102) = 9.094, *p* < 0.001 (η^2^ = 0.211); specifically, within the distant condition accuracy was higher at 100 mg versus Placebo *t*(34) = 3.891, *p* < 0.001 (*d* = 0.658), 200 mg versus Placebo *t*(34) = 3.490, *p* < 0.01 (*d* = 0.590), and 400 mg versus Placebo *t*(34) = 4.708, *p* < 0.001 (*d* = 0.798). This same effect was not found in the proximal condition (*p*’s > 0.58). These results replicate the symbolic distance effect and show that caffeine promotes the global spatial processing bias beginning at 100 mg caffeine intake.

No effects were found for response time (*p*’s > 0.70).

### Experiment 2: Habitual caffeine consumers

Map learning task and spatial description task data reflect 32 complete data sets, as one subject failed to complete tasks during the 200 mg dose test session, and one other subject failed to complete tasks during the 400 mg dose test session.

#### Map learning task

Accuracy data replicated earlier results with a main effect of Distance *F*(1, 31) = 35.133, *p* < 0.001 (η^2^ = 0.108), in which accuracy was higher for distant relative to proximal distances (Proximal = 0.70 ± 0.02; Distant = 0.78 ± 03). There was also a Distance × Caffeine interaction *F*(3, 93) = 3.196, *p* < 0.05 (η^2^ = 0.018). As depicted in Table 4, distant distance accuracy rates marginally increased as a function of caffeine dose; specifically, within the distant condition *F*(3, 93) = 2.633, *p* < 0.06, accuracy was not higher at 100 mg (*p* > 0.95) or 200 mg (*p* > 0.13) versus Placebo but was marginally accuracy at 400 mg versus Placebo *t*(31) = 1.955, *p* < 0.07 (*d* = 0.346). This same effect was not found in the proximal condition (*p’s* > 0.45). These results replicate the symbolic distance effect and show that caffeine exacerbates the effect, only marginally after 400 mg caffeine intake in high habitual caffeine consumers.

**Table 4:** **Map learning task and spatial description task mean accuracy and response time (SE) in high habitual caffeine consumers (*n* = 36)**.

		Map learning task				Spatial descriptions task			
		Proximal		Distal		Proximal		Distal	
		M	SE	M	SE	M	SE	M	SE
Accuracy	0 mg	0.70	0.03	0.74	0.03	0.73	0.03	0.75	0.04
	100 mg	0.69	0.03	0.75	0.03	0.67	0.04	0.74	0.04
	200 mg	0.73	0.03	0.80	0.03	0.70	0.03	0.75	0.03
	400 mg	0.67	0.03	0.81	0.03	0.65	0.04	0.72	0.03
Response time	0 mg	3254.28	275.64	2790.53	230.40	2701.23	240.52	2793.07	240.49
	100 mg	3238.95	236.98	2437.73	203.94	2678.42	199.98	2706.29	196.47
	200 mg	3375.27	240.27	2731.90	208.05	2662.35	205.99	2787.82	235.65
	400 mg	3340.47	221.65	2783.42	190.40	2447.75	218.53	2642.49	230.92

The table represents proximal relative to distant distal comparisons for each of the four Caffeine doses (0, 100, 200, 400 mg). In the map learning task, distal distance accuracy rates marginally increased as a function of caffeine dose; specifically, within the distant condition accuracy was not higher at 100 mg versus Placebo, but showed marginally higher accuracy at 200 mg versus Placebo (*p* < 0.10), and 400 mg versus Placebo (*p* < 0.07). This same effect was not found in the proximal condition. No effects were found for the spatial description task.

Analysis of response time showed main effects for Distance *F*(1, 31) = 103.521, *p* < 0.001 (η^2^ = 0.184), in which response time was higher for distant relative to proximal distances, again replicating the symbolic distance effect (Proximal = 3310.02 ± 202.66 ms; Distant = 2688.57 ± 176.03 ms). No effects of Caffeine or interactions were found for response time.

#### Spatial description task

Accuracy data replicated earlier results with a main effect of Distance *F*(1, 33) = 28.830, *p* < 0.001 (η^2^ = 0.039), in which accuracy was higher for distant relative to proximal distances, demonstrating greater accuracy for landmarks that are farther apart than closer together (Proximal = 0.69 ± 0.03; Distant = 0.74 ± 0.03). No effects of Caffeine or interactions were found for accuracy (*p*’s > 0.28).

No effects were found for response time (*p*’s > 0.10).

### Comparing high and low consumers

To specifically test differences across consumption profiles, we calculated change scores by subtracting the mean accuracy proximal from distant distal comparisons for both the Map Learning and spatial descriptions tasks. Analysis of the map learning task replicated findings of Experiments 1 and 2, finding a main effect of Caffeine *F*(3, 198) = 6.692, *p* < 0.001 (η^2^ = 0.084), such that the difference in accuracy between distant and proximal distances was not higher at 100 mg versus Placebo (*p* > 0.43), but was higher at 200 mg versus Placebo *t*(68) = 3.056, *p* < 0.01 (*d* = 0.368) and 400 mg versus Placebo *t*(67) = 4.102, *p* < 0.001 (*d* = 0.497). These results show that caffeine exacerbates the global processing bias beginning at 200 mg caffeine intake across habitual consumption profiles.

**Figure 1:**
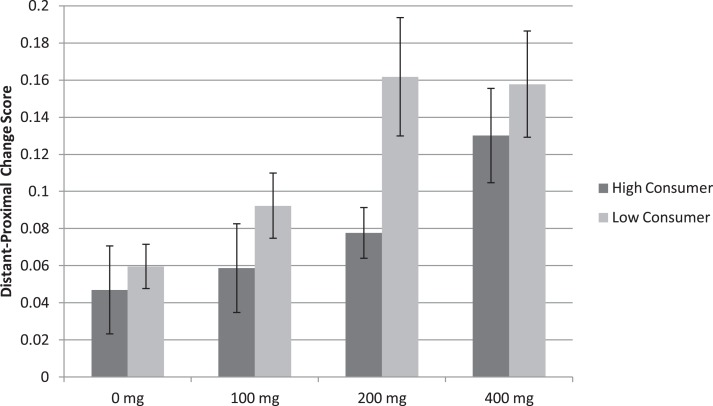
**Map learning task mean accuracy (SE) in both low and high habitual consumers (*n* = 70)**. The graph represents change scores for distant minus proximal distal comparisons. Accuracy was higher in low relative to high habitual caffeine consumers *(p* < 0.05).

**Figure 2:**
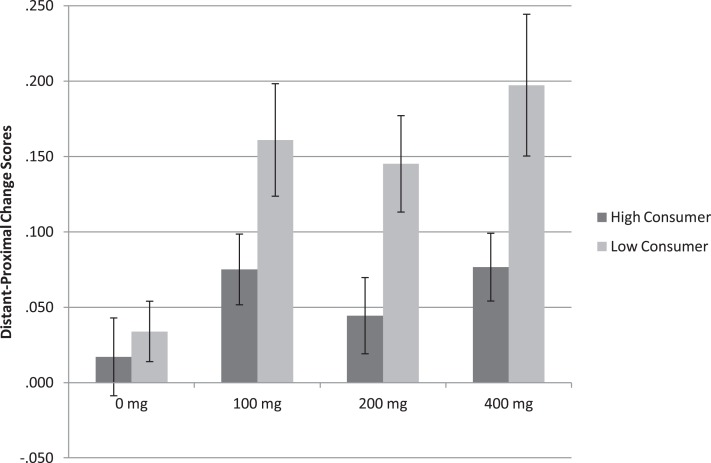
**Spatial description task mean accuracy (SE) in both low and high habitual consumers (*n* = 70)**. The graph represents change scores for distant minus proximal distal comparisons. The difference in accuracy between distant and proximal distances was higher after all doses caffeine intake than placebo (*p* < 0.001) and higher in low relative to high habitual caffeine consumers (*p* < 0.001).

As depicted in Figure 1, a main effect of Consumption profile *F*(1, 66) = 4.54, *p* < 0.05 (η^2^ = 0.244) showed that the difference in accuracy between distant and proximal distances was higher in low relative to high habitual caffeine consumers (Low = 0.12 ± 0.01; High = 0.08 ± 0.01; Figure 2). This finding indicates that at relevant doses, caffeine has greater effects on spatial processing in low relative to high habitual caffeine consumers. No Caffeine × Consumption interaction was found (*p* > 0.54).

Analysis of the spatial description task replicated findings of Experiments 1 and 2, showing a main effect of Caffeine *F*(3, 201) = 4.652, *p* < 0.001 (η^2^ = 0.060), such that the difference in accuracy between distant and proximal distances was higher after 100 mg *t*(68) = 3.045, *p* < 0.01 (*d* = 0.366), 200 mg *t*(68) = 2.567, *p* < 0.05 (*d* = 0.309), and 400 mg caffeine *t*(68) = 3.697, *p* < 0.001 (*d* = 0.445) than placebo. As depicted in Figure 2, a main effect of Consumption profile *F*(1, 67) = 19.427, *p* < 0.001 (η^2^ = 0.122) showed that accuracy was higher in low relative to high habitual caffeine consumers (Low = 0.13 ± 0.01; High = 0.05 ± 0.01), again showing that caffeine influences spatial processing more so in low than high habitual caffeine consumers. No Caffeine × Consumption interaction was found (*p* > 0.39).

## Discussion

We evaluated the influence of caffeine on proximal and distant landmark comparisons in low habitual (Experiment 1) and high habitual (Experiment 2) caffeine consumers. We used two tasks in which subjects studied either maps or spatial descriptions of environments and later completed spatial statement verification tasks that related landmark pairs that were either close (proximal) or far (distal) from one another. Across both experiments and tasks, we replicated previous findings that accuracy is higher for comparisons between landmarks that are farther apart relative to closer together, i.e., the symbolic distance effect (Moyer and Bayer, 1976; Navon, 1977). On the map learning task, caffeine enhanced memory for distal (i.e., global) compared to proximal (i.e., local) comparisons at 100 (marginal), 200, and 400 mg caffeine in non-habitual consumers, and marginally beginning at 400 mg caffeine in habitual consumers. On the spatial descriptions task, caffeine enhanced memory for distal compared to proximal comparisons beginning at 100 mg in non-habitual but not habitual consumers. These findings support extant evidence that caffeine-induced physiological arousal amplifies global spatial processing biases, and these effects are at least partially driven by participants’ caffeine consumption levels. Critically, our results are unique to physiological arousal, as we showed no reliable evidence that caffeine influenced rated pleasant or unpleasant mood.

### Caffeine increases global focus in spatial memory

We found only marginal effects of caffeine to support our first hypothesis, i.e., the arousal states produced via caffeine administration would influence participants’ ability to accurately memorize spatial information. Such effects were found in the spatial descriptions task, in which 200 and 400 mg caffeine marginally improved accuracy relative to placebo in low habitual caffeine consumers. However the data support our second hypothesis that increasing doses of caffeine would induce an increasingly global focus in spatial memory. The results are in line with previous findings that emotional arousal strengthens the representation of distal spatial relationships (Brunyé et al., 2009) and extend these findings by providing evidence that physiological arousal devoid of any valence manipulation, as induced by caffeine, accentuates the global processing bias. The results also support data showing that caffeine augments the global processing bias using hierarchical visual attention (Mahoney et al., 2011) and language-based materials (Brunyé et al., 2012). The results extend such extant findings in several ways. Mahoney and colleagues evaluated the influence of a range of caffeine doses, i.e., 0, 100, 200, and 400 mg caffeine as in the present study, on the Hierarchical Shape and Hierarchical Letter Tasks, which are compound stimuli tasks (i.e., smaller stimuli made of larger stimuli; Navon, 2003) in low habitual caffeine consumers. Although the present study is similar in design and shows a similar global processing accentuation to Mahoney et al. (2011), it goes further in demonstrating that the effect applies during tasks involving both spatial perception (i.e., perceiving the map) and spatial memory (i.e., representing then retrieving the map). Furthermore, we extend the literature to a relatively ecologically relevant task that involves spatial processes demanded on a daily basis such as during navigation through familiar or unfamiliar environments. Additionally, the present findings indicate that at similar doses, caffeine augments global processing to a greater extent in low relative to high habitual caffeine consumers.

Caffeine’s influence on global and local spatial representations may stem, in part, from up-regulation of norepinephrine and serotonin activity. Caffeine is a non-selective competitive adenosine receptor antagonist which exerts its effects primarily through adenosine A_1_ and A_2A_ receptors (Ferre, 2010). Adenosine, in turn, influences other central ascending neurotransmitter systems, including the dopaminergic, noradrenergic, and acetylcholinergic systems. Normally, endogenous adenosine inhibits neurotransmission but caffeine blocks this inhibition, thus increasing extracellular dopamine, noradrenalin, and acetylcholine concentrations (Ferre et al., 1997; Koppelstaetter et al., 2010). Caffeine increases resting-state arousal (Barry et al., 2005), and, though tentative, arousal may be associated with greater right than left hemisphere activity (Nitschke et al., 1999).

Several decades of work suggest a right-hemisphere advantage for global processing, and there is some suggestion of increased right-hemisphere activity during physiological arousal-induced via exercise or caffeine administration. Patients with left versus right-hemisphere neurological lesions tend to show impaired local and global perceptual processing, respectively (Robertson et al., 1988; Christie et al., 2012). In healthy adults, studies using event-related potential (ERP) show right-hemisphere dominance for global, and left for local, visual attention (Heinze and Munte, 1993; Proverbio et al., 1998; Yamaguchi et al., 2000). The right-hemisphere is also more active than the left during states of physiological arousal, such as during aerobic exercise (Woo et al., 2009). Evidence specifically regarding caffeine’s selective influence on brain activation and relative hemispheric activity is very limited. Whereas a few studies suggest increased right versus left activity following caffeine consumption (Lorist and Snel, 1997; Koppelstaetter et al., 2008), some suggest no hemispheric differences (Kennedy and Haskell, 2011), and others suggest increased left versus right activity (Kuchinke and Lux, 2012). Overall, caffeine may upregulate levels of brain dopamine, serotonin, and norepinephrine, neurotransmitter systems that appear to be at least partially lateralized to the right-hemisphere (Oke et al., 1978, 1980; Arato et al., 1991; Davidson et al., 2004; Smith et al., 2006b). The right-hemisphere has also been implicated in the processing and representation of spatial information including relative landmark locations (Smith et al., 1996; Bohbot et al., 1998). Overlapping right-hemisphere neural mechanisms engaged during global processing, spatial cognition, and states of physiological arousal may prove responsible for the present results; future work might directly consider this possibility by complementing our design with functional neuroimaging.

### Caffeine accentuates global processing in both high and low caffeine consumers

To specifically test differences across consumption profiles, we calculated change scores by subtracting the mean accuracy proximal from distant distal comparisons and compared these scores across the two consumption profiles. We found that the difference between low and high accuracy levels was higher in low relative to high habitual caffeine consumers in both tasks, but found no interactions with dose. Thus we support the final hypothesis that the influence of caffeine on spatial representation will be greater in low versus high consumers by showing greater difference in accuracy between distant and proximal distal comparisons in individuals who do not typically consume caffeine.

The argument over whether caffeine influences cognition or merely reverses withdrawal effects is ongoing. Although this is the first study to compare the impact of caffeine on memory for spatial relationships, previous studies assessing caffeine and other types of memory provide conflicting reports, e.g., caffeine improved working memory regardless of habitual consumption profile in one study (Addicott and Laurienti, 2009) but neither caffeine, habitual caffeine consumption, nor caffeine withdrawal influenced working memory, short-term, or delayed memory in other studies (Mitchell and Redman, 1992; Hewlett and Smith, 2007; Koppelstaetter et al., 2008).

In order to better determine whether caffeine’s influence on spatial processing is influenced by caffeine withdrawal, future studies should employ a “normal caffeine consumption” condition, which could be compared to the placebo condition. Regardless of this limitation to our study design, comparing the high and low caffeine consumers’ results, separately, indicates that caffeine enhances the global processing bias in both habitual and non-habitual caffeine consumers, and that a larger caffeine dose is necessary to achieve the same effect in high habitual caffeine consumers, in that for distal landmark comparisons, caffeine improved accuracy beginning between 100 mg (map learning task) and 200 mg (spatial descriptions task) in low habitual consumers, but only marginally improved accuracy at 400 mg in high habitual caffeine consumers.

Comparisons between high and low habitual caffeine consumers provide convincing evidence that caffeine accentuates global processing across habitual consumption profiles. These findings refute the contention that caffeine’s effects are primarily due to reversal of caffeine withdrawal, as past work has found that caffeine abstinence impairs cognitive performance and caffeine intake does not reverse withdrawal effects (Rogers et al., 2005). However, the present results indicate that caffeine promotes the global processing bias across habitual consumption profiles, after as little as 200 mg caffeine intake. The role of caffeine withdrawal in caffeine-induced changes to cognitive performance may be domain-dependent, in that caffeine enhanced working memory following caffeine abstinence and normal consumption but improved psychomotor performance following caffeine abstinence only (Addicott and Laurienti, 2009), and caffeine enhances executive function in both habitual and non-habitual caffeine consumers (Brunyé et al., 2010a,b). Thus, habitual consumption profiles may play a role in caffeine’s influence on psychomotor performance, but not on other cognitive domains, including global spatial processing.

However, we found no interaction between Caffeine dose and Consumption profile on the global processing bias (i.e., difference in accuracy for distal minus proximal landmark comparisons), which indicates that caffeine did not differentially influence accuracy for distal and proximal comparisons between low and high habitual caffeine consumers in a dose-dependent manner with the range of doses administered. Further, we found no main effects or interactions between caffeine and distance on the spatial descriptions task in high habitual caffeine consumers, which leaves open the question of whether a higher dose is necessary to see the global processing bias in habitual caffeine consumers or, conversely, whether caffeine does not influence spatial processing on this task in individuals who regularly consume caffeine.

### Conclusion

In summary, caffeine amplified a globally focused spatial representation after as little as 100 mg caffeine, less than the amount of caffeine in Starbucks 12 oz brewed coffee (260 mg). The results have implications in everyday life, as daily caffeine intake could potentially lead to more global spatial representations. A morning cup of coffee, for example, could enhance memory for the general location of key landmarks or regions (e.g., theater district, Empire State Building), but perhaps at the expense of knowledge (e.g., particular theaters, buildings adjacent to the Empire State Building).

The globally focused spatial representation is evident in both habitual and non-habitual consumers, although it appears to require more caffeine to achieve the same global bias in habitual consumers. Thus the caffeine’s influence on spatial representation and memory may be vulnerable to influences of caffeine tolerance and withdrawal. Thus an increasingly large cup of coffee may be needed for enhanced memory for global environmental features.

## Conflict of Interest Statement

The authors declare that the research was conducted in the absence of any commercial or financial relationships that could be construed as a potential conflict of interest.
